# Real-World Effectiveness and Safety of Esketamine Nasal Spray in Treatment-Resistant Depression: A Retrospective Observational Study

**DOI:** 10.3390/healthcare14172717

**Published:** 2026-08-26

**Authors:** Mostafa A. Sayed Ali, Palanisamy Amirthalingam, Hanan Alshareef, Mohammed Zayed Alassiry, Ahmed Samir Elshenawy, Sayed Hosam Eldin Mansour, Ahmed Aljabri

**Affiliations:** 1 Department of Pharmacy Practice, Faculty of Pharmacy, University of Tabuk, Tabuk 47491, Saudi Arabia; pchettiar@ut.edu.sa (P.A.); halsharef@ut.edu.sa (H.A.); 2 Department of Psychiatry, Eradah Mental Health Hospital, Aseer Health Cluster, Abha 62585, Saudi Arabia; mal-asiri@moh.gov.sa (M.Z.A.); amohamed75@moh.gov.sa (A.S.E.); sewais@moh.gov.sa (S.H.E.M.); 3 Department of Pharmacy Practice, Faculty of Pharmacy, King Abdulaziz University, Jeddah 21589, Saudi Arabia; amaljabri@kau.edu.sa

**Keywords:** effectiveness, esketamine, major depressive disorder, treatment-resistant, venlafaxine

## Abstract

**Highlights:**

**What are the main findings?**

- In routine Saudi psychiatric care, intranasal esketamine was preferentially prescribed to patients with more severe depression and a greater number of previous antidepressant failures.
- In propensity-score matched analyses, follow-up PHQ-9 scores were modestly lower in the esketamine group than in the venlafaxine group; the estimated differences were 1.02 points at 3 months and 1.94 points at 6 months.

**What are the implications of the main findings?**

- Observed differences should be interpreted cautiously because treatment allocation was not randomized and follow-up outcomes were available only for patients retained in care.
- The absence of remission in either cohort emphasizes the continuing need for multimodal treatment pathways for treatment-resistant depression.

**Abstract:**

**Background/Objectives**: This study evaluated the real-world effectiveness and safety of intranasal esketamine plus oral antidepressants compared with venlafaxine-based oral antidepressant therapy in adults with moderate-to-severe treatment-resistant depression (TRD). **Methods**: This retrospective cohort study used electronic medical records from a mental health hospital between January 2023 and December 2025. Adults with moderately severe or severe major depressive disorder who had not responded adequately to at least two antidepressant trials received esketamine plus oral antidepressants or venlafaxine-based therapy. The outcomes were changes in the Patient Health Questionnaire-9 (PHQ-9) score, remission, response, minimal clinically important difference (MCID) without response or remission, and documented adverse events at 28 days, 3 months, and 6 months. Propensity score matching was used for continuous follow-up of the PHQ-9 outcomes. **Results**: The analytic baseline cohort included 82 patients treated with intranasal esketamine-based therapy and 87 treated with venlafaxine-based therapy. The esketamine group had greater baseline depression severity and more previous antidepressant failures. At 3 months, no patients who continued treatment in either group met the remission criterion, one esketamine-treated patient achieved a response, and 37/61 (60.7%) esketamine-treated patients and 23/72 (31.9%) venlafaxine-treated patients achieved MCID without response or remission. At 6 months, remission remained absent; 27/61 (44.3%) esketamine-treated patients and 9/72 (12.5%) venlafaxine-treated patients met the response criterion, whereas 34/61 (55.7%) and 63/72 (87.5%) achieved MCID without response or remission, respectively. In propensity score-matched analyses, follow-up PHQ-9 scores were 1.02 points lower at 3 months (average treatment effect [ATE], −1.02; 95% CI, −1.70 to −0.35; *p* = 0.003) and 1.94 points lower at 6 months (ATE, −1.94; 95% CI, −3.17 to −0.71; *p* = 0.002) in the esketamine group than in the venlafaxine group. Dissociation and dizziness were documented more often in the esketamine group, whereas sexual dysfunction was documented more often in the venlafaxine group. **Conclusions**: In this retrospective routine care cohort, esketamine was used in patients with more complex TRD and was associated with modestly lower matched follow-up PHQ-9 scores. The observational design, baseline imbalance, and attrition precluded causal conclusions.

## 1. Introduction

Depressive disorders remain a major contributor to disability and health loss worldwide. The Global Burden of Disease (GBD) 2023 Study identified depressive and anxiety disorders among the noncommunicable conditions with increasing age-standardized disability-adjusted life years between 2013 and 2023. These findings underscore the continuing global burden of common mental disorders [1]. In Saudi Arabia, depression has also been identified as a leading contributor to disability, particularly among women [2].

Globally, approximately 20–40% of individuals diagnosed with MDD develop treatment-resistant depression (TRD), commonly defined as failure to achieve an adequate response or remission after at least two antidepressant trials of adequate dose and duration during the current depressive episode [3,4,5]. The burden of MDD in Gulf Cooperation Council (GCC) countries is substantial, with an estimated 800–1100 disability-adjusted life years per 100,000 inhabitants. Its prevalence has also increased markedly since the onset of the COVID-19 pandemic [6]. In Saudi Arabia, TRD imposes a considerable clinical and economic burden, with its direct economic cost estimated at SAR 14,997 million [7].

Guideline-recommended treatment strategies emphasize the collaborative, personalized, and systematic management of TRD [8]. Pharmacologic approaches remain the cornerstone of treatment and include switching to an alternative antidepressant, combining two antidepressants, or augmenting antidepressant therapy with agents such as atypical antipsychotics or lithium [9]. Non-pharmacological interventions such as cognitive behavioral therapy and brain stimulation techniques (e.g., transcranial magnetic stimulation, or electroconvulsive therapy) are also commonly recommended [8,10]. Despite these treatment approaches, TRD remains a major clinical challenge [11]. Standard pharmacological, psychotherapeutic, and neurostimulation treatments yield low response and remission rates in patients with TRD. Moreover, patients who achieve response or remission experience relapse within six months [12]. Compared with individuals with non-treatment-resistant MDD, those with TRD are more likely to experience suicidal ideation and greater impairment in work productivity, daily activities, and healthcare utilization [12,13,14].

Augmentation therapy with atypical antipsychotic medications, particularly aripiprazole and brexpiprazole, has demonstrated moderate effectiveness in enhancing response rates in patients with TRD [15]. However, these treatments may be limited by adverse effects, and lithium requires careful monitoring because of its narrow therapeutic index and potential toxicity. Therefore, additional treatment options are required.

Esketamine, the S-enantiomer of ketamine, administered as a nasal spray in combination with an oral antidepressant, represents a potential breakthrough in the treatment of TRD [16]. It has been approved by the US Food and Drug Administration and European Medicines Agency along with a selective serotonin reuptake inhibitor (SSRI) or a serotonin-norepinephrine reuptake inhibitor (SNRI) for TRD [17]. In clinical trials, esketamine nasal spray combined with a newly initiated SSRI or SNRI produced greater reductions in depressive symptoms than a placebo nasal spray plus an oral antidepressant in patients with TRD [16,18,19]. Additionally, esketamine nasal spray was more effective in preventing relapse among patients who had achieved stable remission or a stable response [16]. In the ESCAPE-TRD trial, esketamine plus an SSRI or SNRI produced a higher week-8 remission rate than extended-release quetiapine plus an SSRI or SNRI (27.1% vs. 17.6%) [20].

These studies have demonstrated the rapid antidepressant effects of esketamine, with some evidence supporting its ability to reduce suicidal ideation. However, concerns remain regarding its potential for abuse, cognitive side effects, and long-term efficacy. Because of the risks of serious adverse events, esketamine must be administered in a certified healthcare setting under direct supervision of a healthcare provider. Further research is necessary to validate these findings in diverse populations. Moreover, Saudi real-world data comparing esketamine-based treatment with established oral antidepressant strategies remain limited. The available Saudi literature primarily focuses on the clinical and economic burden of TRD, whereas esketamine-specific evidence is sparse [7]. Regional publications have mainly provided expert recommendations on the implementation of esketamine services in Gulf Cooperation Council countries, and emerging Saudi research has explored mental-health professionals’ perspectives on its use [21]. However, published Saudi data describing real-world prescribing patterns, including comparative routine care evidence assessing intranasal esketamine plus an oral antidepressant against established oral antidepressant strategies, are lacking.

Therefore, this retrospective cohort study aimed to provide real-world evidence on the effectiveness and safety profile of intranasal esketamine plus an oral antidepressant compared with venlafaxine-based oral antidepressant therapy among adults with moderate-to-severe TRD treated at a Saudi mental-health hospital.

## 2. Materials and Methods

### 2.1. Study Design and Setting

This retrospective cohort study used electronic medical records (EMRs) from the Eradah Mental Health Hospital, Aseer Health Cluster, Saudi Arabia. Records from 1 January 2023 to 31 December 2025, were reviewed. The study design and manuscript preparation followed the Strengthening the Reporting of Observational Studies in Epidemiology (STROBE) guidelines for cohort studies [22].

### 2.2. Participants and Treatment Groups

Eligible participants were adults aged 18–75 years with moderate-to-severe TRD who met the Diagnostic and Statistical Manual of Mental Disorders (DSM-5-TR) criteria for MDD. Psychiatrists initially screened eligible patients to assess the frequency and severity of depressive symptoms using the Patient Health Questionnaire-9 (PHQ-9) depression severity scale. Moderately severe and severe depression were defined as PHQ-9 scores of 15–19 and 20–27, respectively. TRD was defined as an inadequate response to at least two or more different ADs of two different classes (typically less than a 25–50% reduction), administered at an adequate dose and duration (usually 6–8 weeks per trial) during the current depressive episode [23]. Patients were eligible when they had not responded adequately to these trials and were unable to receive ECT due to contraindication, no access, resistance or patient refusal. Patients were excluded if they had a history of substance abuse or a primary diagnosis of major psychiatric disorders, such as bipolar or personality disorders. Pregnant or breastfeeding females and patients with uncontrolled hypertension or cardiovascular diseases were also excluded.

Eligible patients receiving intranasal esketamine plus oral antidepressants (esketamine group) were compared with patients receiving venlafaxine-based oral antidepressant therapy (venlafaxine group). Esketamine treatment was initiated using a planned twice-weekly intranasal induction schedule over 4 weeks. The initial prescribed dose was 56 mg or 84 mg, selected by the treating psychiatrist according to clinical judgment. Esketamine was administered in the hospital under direct healthcare-provider supervision. Patients who continued treatment after induction entered a continuation/maintenance phase of up to 24 weeks, during which administration frequency was generally reduced to once weekly, with selected patients receiving treatment every 2 weeks according to clinical response and tolerability.

Electronic medical records were reviewed to identify the planned treatment schedule, initial prescribed dose, documented discontinuation, and entry into continuation treatment. The records did not provide complete standardized patient-level data on cumulative administrations, cumulative dose, exact treatment duration, or maintenance dosing frequency throughout follow-up.

Venlafaxine was initiated at 75 mg once daily, increased to 150 mg after 2 weeks as clinically indicated, and maintained or increased to 225 mg according to response and tolerability. Baseline concomitant psychotropic medication classes were recorded; however, adherence to oral antidepressants, specific time-varying oral antidepressant regimens, and changes in concurrent psychotropic medications during follow-up could not be reliably assessed.

### 2.3. Sample Size Calculation

The sample size was calculated for the primary comparative outcome, mean change in the PHQ-9 score from baseline to 3 months, using a two-sided independent-samples comparison of means. Assuming a clinically relevant between-group difference of 3 PHQ-9 points, a common standard deviation of 6 points, a two-sided alpha level of 0.05, 80% power, and equal allocation between treatment groups, the required sample size was calculated as follows [24]:

$$n_{\mathrm{per}\;\mathrm{group}}=\frac{2(Z_{1-\alpha /2}+Z_{1-\beta }{)}^{2}\sigma^{2}}{\Delta^{2}}$$

$$n_{\mathrm{per}\;\mathrm{group}}=\frac{2(1.96+0.84{)}^{2}(6{)}^{2}}{(3{)}^{2}}\approx 63$$

Accordingly, the target sample size was approximately 63 participants per group.

### 2.4. Data Collection and Management

Data extracted from the EMRs included demographic characteristics (age, sex, ethnicity, body mass index (BMI), education, employment, marital status, and smoking status) and clinical characteristics (duration of disease, family history of MDD, baseline PHQ-9 severity category, number of previous antidepressant treatment failures, and concurrent psychotropic medications).

The number of previous antidepressant failures was categorized as 2, 3–4, 5–6, 7–8, and 9–10 failed treatments. Concurrent medication use was classified according to the therapeutic class, including SSRIs, SNRIs, tricyclic antidepressants, atypical antidepressants, antipsychotics, benzodiazepines, and mood stabilizers. These data were used to characterize the treatment complexity in both cohorts and to adjust the comparative analysis for the measured baseline differences.

PHQ-9 scores were recorded at baseline and at 3- and 6-month follow-up assessments. The 28-day induction phase was assessed for treatment continuation, treatment discontinuation, and documented reasons for discontinuation. Reasons for discontinuation were extracted from the EMR and categorized as failure or no response, patient preference, or adverse effects.

### 2.5. Outcome Definition

**Figure 1:**
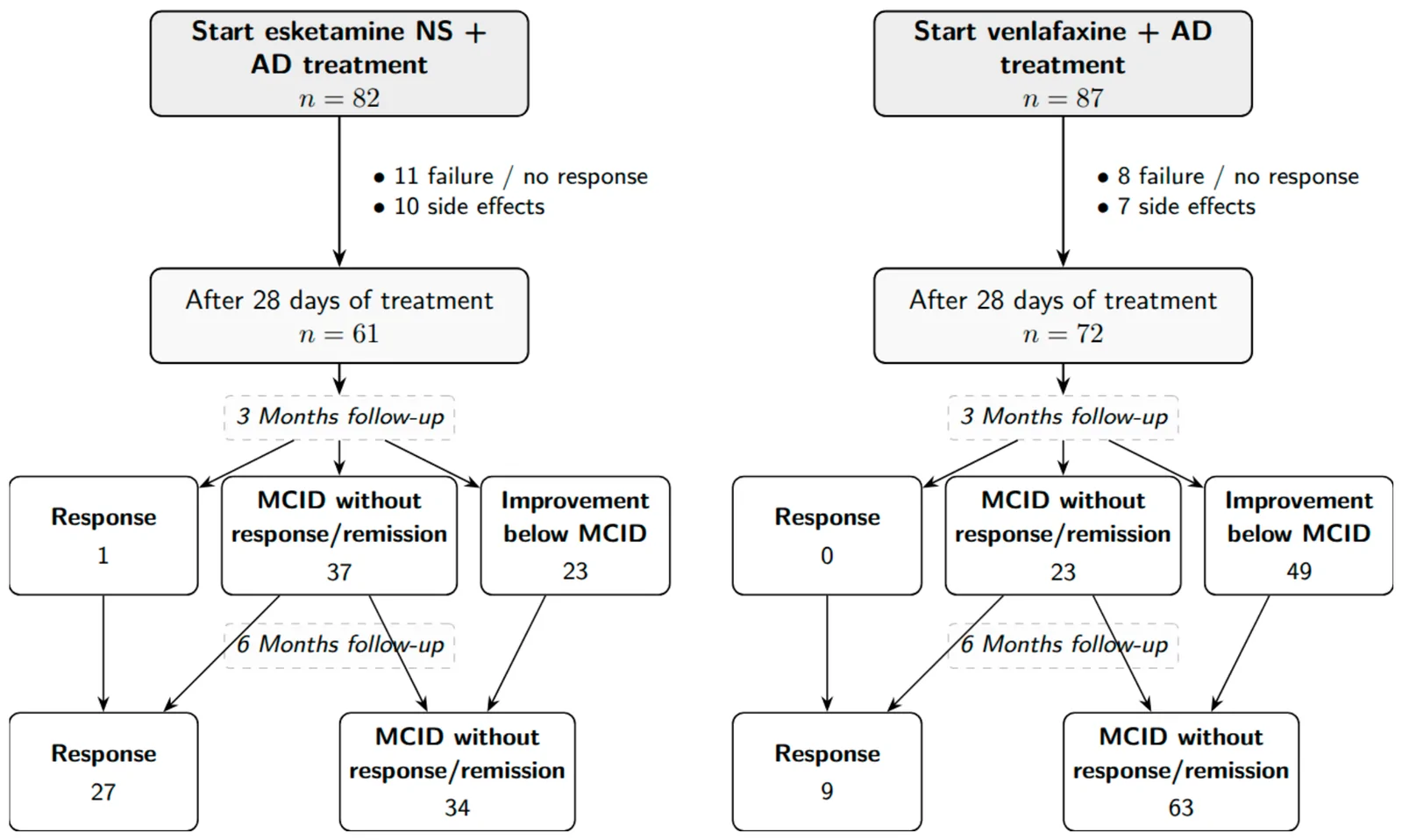
Flowchart of patient inclusion and treatment outcomes for intranasal esketamine plus oral antidepressant therapy and venlafaxine-based oral antidepressant therapy. Clinical outcomes were classified hierarchically and were mutually exclusive. Remission was defined as a follow-up PHQ-9 score < 5. Response was defined as a ≥50% reduction from baseline PHQ-9 score with a follow-up score < 10. MCID was defined as an absolute PHQ-9 reduction of ≥5 points from baseline among patients who did not meet response or remission criteria. Patients classified as “Improvement below MCID” had a reduction of <5 points, no change, or worsening in PHQ-9 score. AD, antidepressant; Esketamine NS, esketamine nasal spray; MCID, minimal clinically important difference.

The primary clinical effectiveness measure was the change in PHQ-9 score from baseline. The PHQ-9 is a patient-reported measure used to assess depression severity and monitor treatment responses. The original PHQ-9 validation work by Kroenke et al. explicitly positions it as suitable for “selecting and monitoring treatment,” [25]. An individual-participant data meta-analysis confirmed the accuracy of the PHQ-9 for detecting MDD in primary care and research settings, supporting its use as an outcome measure in pharmacotherapy studies [26]. Multiple validation studies have reported strong psychometric properties of the Arabic version of the Patient Health Questionnaire-9 (PHQ-9) including a Saudi study by AlHadi et al. [27]. Response was defined as at least 50.0% reduction in the PHQ-9 total score and follow-up PHQ-9 < 10; remission was defined as a PHQ-9 total score < 5 and the Minimal Clinically Important Difference (MCID) was defined as an absolute reduction of at least 5 points from the baseline PHQ-9 score without response or remission [25,28]. Patients classified as “improvement below MCID” had a reduction of <5 points, no change, or worsening in PHQ-9 score. Clinical outcomes were assessed at 28 days, 3 months, and 6 months. At 3 and 6 months, patients with documented follow-up PHQ-9 scores were classified hierarchically and mutually exclusively as having remission, response, MCID without response or remission, or improvement below the MCID (Figure 1).

Safety outcomes included adverse events documented in the electronic medical record as serious, and discontinuations attributed to adverse events. The classification of an event as serious was based on routine clinical documentation. Adverse events were identified from routine EMR documentation and were summarized as frequencies. Therefore, the safety analysis described recorded clinical events and did not estimate the comparative adverse-event incidence or establish causality.

### 2.6. Statistical Analysis

Statistical analyses were performed using Stata software, version 19, StataCorp LLC, College Station, TX, USA. Baseline demographic and clinical characteristics were summarized using descriptive statistics. Categorical variables were reported as frequencies and percentages and compared between groups using chi-square tests or Fisher’s exact tests, as appropriate. Continuous variables were summarized as means and standard deviations or medians and ranges, as appropriate. Independent-samples *t* test was used to compare the mean ages between the two treatment groups. The Mann–Whitney U test was used to compare non-normally distributed continuous variables, such as BMI and duration of disease. Standardized mean differences (SMDs) were calculated to quantify the baseline imbalance between the groups.

Because treatment allocation was not randomized, propensity score matching was performed separately for the continuous follow-up PHQ-9 outcomes at 3 and 6 months. For each outcome-specific complete-case sample, propensity scores were estimated using a logistic regression model including sex, age, BMI, family history of MDD, employment status, smoking status, marital status, educational level, duration of disease, baseline PHQ-9 score, number of failed antidepressant treatments and concurrent psychotropic medication. Stata version 19 (teffects psmatch) was used to estimate the average treatment effect (ATE) using 1:1 nearest-neighbor propensity score matching with replacement (ate nneighbor (1)). No caliper, trimming, or additional common-support restriction was specified. The analysis was based on 133 unique patients with observed outcomes and matching with replacement permitted repeated use of comparator observations, and thus some control observations could be reused. Covariate balance was assessed using standardized mean differences (SMDs) before and after matching, with an absolute SMD < 0.10 considered an acceptable balance. The matched analysis estimated the ATE, expressed as the mean difference in follow-up PHQ-9 scores between treatment groups. A negative ATE indicated a lower follow-up PHQ-9 score in the esketamine group.

All tests were two-sided, and a *p*-value < 0.05 was considered statistically significant. Unadjusted categorical outcome analyses were interpreted descriptively because of baseline imbalance, nonrandom treatment allocation, and follow-up attrition.

## 3. Results

### 3.1. Baseline Patient Characteristics

A total of 169 patients were included in the comparative analysis, comprising 82 patients treated with intranasal esketamine plus oral antidepressants and 87 patients who received venlafaxine-based oral antidepressant therapy (Figure 1). The mean age was similar between the esketamine and venlafaxine groups (37.1 [SD, 10.5] vs. 36.6 [SD, 11.7] years; *p* = 0.786). The mean duration of depression was also similar at 7.8 years in each group (*p* = 0.384) (Table 1).

Several baseline characteristics differed between the groups. Males accounted for a greater proportion of the esketamine group than the venlafaxine group (76.8% vs. 60.9%; *p* = 0.026). The education level also differed between groups (*p* = 0.001): 43.9% of esketamine-treated patients had a university degree, compared with 19.5% of venlafaxine-treated patients. Baseline depressive symptom severity was higher in the esketamine group. Severe depression (PHQ-9 score, 20–27) was recorded for 63.4% of esketamine-treated patients and 34.5% of venlafaxine-treated patients; moderately severe depression (PHQ-9 score, 15–19) was recorded for 36.6% and 65.5%, respectively (*p* = 0.001).

The esketamine group also had a more extensive history of non-response to antidepressants (*p* = 0.001). In this group, 43.9% of patients had failed 5–6 previous antidepressant treatments and 23.2% had failed 7–8 antidepressant treatments. In contrast, patients in the venlafaxine group were concentrated in the 1–2 and 3–4 failed-antidepressant categories; 4.6% had failed 7–8 antidepressant treatments, and none had failed more than eight.

Concurrent psychotropic medication use was common in both groups. In the esketamine group, the most frequently documented medication classes were SNRIs (62.2%), antipsychotics (61.0%), SSRIs (52.4%), atypical antidepressants (24.4%), and TCAs (17.1%). In the venlafaxine group, SNRIs (73.3%), antipsychotics (62.1%), SSRIs (36.8%), atypical antidepressants (26.4%), and TCAs (20.7%) were documented. These findings describe complex pharmacotherapy in both cohorts, consistent with difficult-to-treat depression in routine clinical practice.

The standardized mean differences (SMDs) in Table 1 indicate an imbalance in the unmatched cohort, particularly for sex, BMI, education, baseline depression severity, number of failed antidepressant treatments, and family history of MDD.

### 3.2. Dosing Patterns

All 82 patients in the esketamine group initiated the planned twice-weekly induction schedule. Of these, 61 entered the documented continuation/maintenance phase. The induction dose was 56 mg in 21 patients (25.7%) and 84 mg in 61 patients (74.3%). During continuation treatment, the administration frequency was reduced to once weekly for most patients, with selected patients receiving treatment every 2 weeks according to clinical response and tolerability. Venlafaxine was initiated at 75 mg once daily, increased to 150 mg after 2 weeks as clinically indicated, and maintained in (56, 64%) or increased to 225 mg (17, 19%) according to response and tolerability (Table 2). Because the retrospective records did not contain a complete standardized record of the total number of administrations, cumulative dose, duration of exposure, or maintenance dosing frequency for each patient, these measures could not be summarized quantitatively. Similarly, longitudinal information on the exact co-administered oral antidepressants and changes in concurrent psychotropic medications was incomplete.

### 3.3. Treatment Outcomes

The baseline cohort comprised 82 patients in the esketamine group and 87 patients in the venlafaxine group. During the 28-day induction phase, treatment was discontinued in 21 esketamine-treated and 15 venlafaxine-treated patients. In both groups, discontinuation was attributed to lack of response or treatment failure in approximately half of cases and to patient preference or adverse events in the remaining cases. Consequently, 61 esketamine-treated and 72 venlafaxine-treated patients entered the documented maintenance phase (Table 3).

At 3 months, the mean change from the baseline PHQ-9 score was −4.73 (95% CI, −5.37 to −4.08) in the esketamine group and −3.41 (95% CI, −3.75 to −3.07) in the venlafaxine group (*p* < 0.001). No patient in either group met the remission criteria (PHQ-9 score < 5). One esketamine-treated patient met the response criterion, and 37/61 (60.7%) esketamine-treated and 23/72 (31.9) venlafaxine-treated patients achieved the MCID without response or remission.

At 6 months, the mean change from the baseline PHQ-9 score was −8.83 (95% CI, −9.76 to −7.90) in the esketamine group and −6.26 (95% CI, −6.87 to −5.64) in the venlafaxine group (*p* < 0.001). No remission was recorded in either group. Response was documented for 27/61 (44.3%) esketamine-treated and 9/72 (12.5%) venlafaxine-treated patients. MCID without response or remission was documented for 34/61 (55.7%) esketamine-treated and 63/72 (87.5%) venlafaxine-treated patients. These proportions should be interpreted in light of differential discontinuation and the descriptive and unadjusted nature of these analyses.

### 3.4. Propensity Scores Matching Analysis

Propensity score matching incorporated sex, age, body mass index, family history of MDD, employment status, smoking status, marital status, educational level, duration of disease, baseline PHQ-9 score, number of failed antidepressant treatments, and concurrent psychotropic medications. In the matched analysis, follow-up PHQ-9 scores at 3 months were, on average, 1.02 points lower in the esketamine group than in the venlafaxine group (ATE, −1.02; 95% CI, −1.70 to −0.35; *p* = 0.003).

At 6 months, matched follow-up PHQ-9 scores were, on average, 1.94 points lower in the esketamine group (ATE, −1.94; 95% CI, −3.17 to −0.71; *p* = 0.002). Although statistically significant, both between-group estimates were smaller than the commonly used 5-point individual-level PHQ-9 MCID threshold.

The balance diagnostics are presented in Supplementary Table S1. An absolute standardized mean difference (SMD) < 0.10 was considered to indicate acceptable balance. At 3 months, matching reduced imbalance for several covariates; however, residual imbalance remained for BMI (SMD = 0.206), number of failed AD (SMD = 0.147), baseline PHQ-9 score (SMD = 0.123), and family history of MDD (SMD = 0.290), the latter being the largest residual imbalance. At 6 months, the largest residual imbalance was for family history of MDD (SMD = 0.249); education level showed a smaller residual imbalance (SMD = 0.171), whereas all remaining covariates had absolute SMDs ≤0.129. Therefore, the propensity-score matched estimates should be interpreted as adjusted associations rather than causal effects.

### 3.5. Safety Profile

The documented adverse-event profiles differed between treatment groups. During the 28-day induction phase, treatment discontinuation attributed to patient preference or adverse effects occurred in 10 of 82 patients (12.2%) receiving esketamine and 7 of 87 patients (8.0%) receiving venlafaxine-based therapy. In the esketamine group, one patient (1.2%) had an event documented as serious, described as severe dissociation. In the venlafaxine group, two patients (2.3%) had events documented as serious; both were described as hypertensive crises (Table 3). No additional adverse events documented as serious were identified during the available continuation and follow-up period.

**Figure 2:**
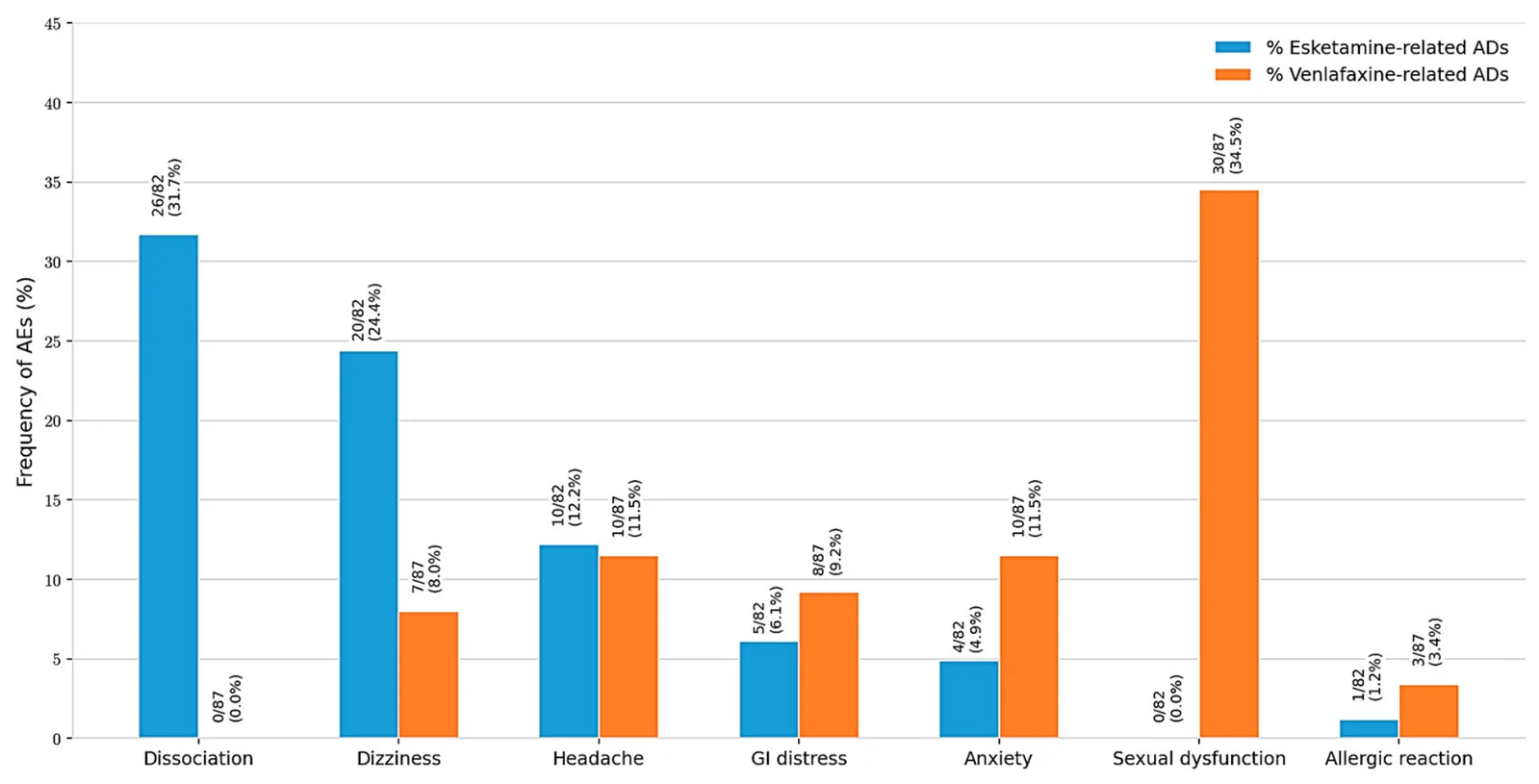
Proportion of patients with documented adverse events during treatment with intranasal esketamine plus oral antidepressants (n = 82) or venlafaxine-based oral antidepressant therapy (n = 87). Bars show the percentage of patients with at least one documented event; data labels show n/N (%). Adverse events were extracted from electronic medical records and were not systematically assessed for severity, duration, or causal relationship to treatment.

Across the induction and continuation phases, dissociation (26/82, 31.7%) and dizziness (20/82, 24.4%) were the most frequently documented adverse events among patients receiving esketamine, followed by headache (10/82, 12.2%) and gastrointestinal symptoms (5/82, 6.1%). No sexual dysfunction was documented in this group. Among patients receiving venlafaxine-based therapy, sexual dysfunction was the most frequently documented adverse event (30/87, 34.5%), followed by headache and anxiety (10/87 each, 11.5%) and gastrointestinal symptoms (8/87, 9.2%). No dissociation was documented in the venlafaxine group (Figure 2). As adverse events were identified from routine electronic medical records, they were not systematically evaluated for onset, duration, severity, dose relationship, or causal attribution. Individual patients may have experienced more than one documented adverse event.

## 4. Discussion

This retrospective observational study compared intranasal esketamine plus oral antidepressants with venlafaxine-based oral antidepressant therapy in adults with moderate-to-severe TRD and a history of antidepressant treatment failure. Patients prescribed esketamine had a higher prevalence of severe baseline depressive symptoms and a greater number of previously failed antidepressant treatments. These characteristics are consistent with a more clinically complex treatment population and are important when interpreting between-group differences.

Baseline imbalance has major implications for interpreting comparative effectiveness. Although the groups had similar mean age and duration of disease, they differed in sex distribution, BMI, educational level, baseline PHQ-9 severity, and extent of previous antidepressant nonresponse. Therefore, unadjusted comparisons should not be interpreted as estimates of treatment effects. In particular, the clinical selection of esketamine for patients with more refractory illness may have influenced both treatment allocation and subsequent outcomes. This pattern is consistent with a common challenge in real-world comparative effectiveness research, where observational treatment groups often differ at treatment initiation, and causal inference requires explicit adjustment for confounding rather than simple descriptive comparison [29].

Both groups showed a reduction in the PHQ-9 scores during follow-up. At 3 months, the mean reduction from baseline was larger in the esketamine group, although the matched between-group difference in the follow-up PHQ-9 score was modest (−1.02 points). At 6 months, the matched difference was −1.94 points. These estimates were statistically significant but smaller than the conventional 5-point individual-level PHQ-9 MCID. The findings indicate a modest, adjusted difference in symptom scores at the group level rather than a clinically meaningful improvement in every individual patient. The residual imbalance, including the modest residual imbalance in baseline PHQ-9 at 3 months, may have biased the corresponding ATE estimate because baseline symptom severity is prognostic of subsequent PHQ-9 scores. Therefore, the propensity-score matched ATEs are interpreted as adjusted associations and not as causal treatment effects. Published work on PHQ-9 changes suggests that clinically meaningful improvement may vary by baseline severity, with average MCID estimates around 3.7 points and larger absolute changes required among patients with more severe baseline symptoms [30]. Some studies have suggested that smaller differences (e.g., 2–3 points) can still be clinically meaningful at the group level, depending on baseline severity especially when persistent over time [31,32].

The absence of documented remission should be considered in the context of randomized and real-world evidence. Differences in baseline severity, prior treatment exposure, outcome measures, treatment adherence, follow-up procedures, and completeness of outcome documentation may account for some of these discrepancies. In randomized TRD trials, esketamine plus an oral antidepressant has shown clinically significant antidepressant effects compared with placebo nasal spray plus oral antidepressant, and continuation treatment has reduced relapse risk among patients who first achieved stable remission or response [19]. In the ESCAPE-TRD trial, esketamine plus an SSRI or SNRI produced higher week-8 remission than quetiapine extended-release plus an SSRI or SNRI, with remission reported in 27.1% versus 17.6% of patients, respectively [20].

The dosing pattern was consistent with standard TRD dosing practices, as most esketamine-treated patients received 84 mg and the reported administration frequency was twice weekly. Regulatory labeling describes twice-weekly dosing during the initial treatment phase and emphasizes that esketamine requires supervised administration because of risks including sedation, dissociation, respiratory depression, abuse, and misuse [33]. The safety findings were consistent with the known, commonly reported adverse events of esketamine, particularly dissociation and dizziness [33]. Sexual dysfunction was most frequently documented in the venlafaxine group. Published evidence has identified sexual dysfunction as a recognized adverse effect of venlafaxine and other SNRIs, including abnormal ejaculation, erectile dysfunction, and reduced libido [34,35]. However, adverse events were identified from routine medical records and were not systematically graded for severity, timing, duration, dose relationship, or contribution to discontinuation. Therefore, the results describe documented safety patterns rather than comparative adverse event risks.

This study used the PHQ-9 to screen the severity of depressive symptoms and as the primary comparative outcome to compare antidepressant effectiveness over time, which is a limitation relative to established clinician-rated scales (e.g., HDRS). Most traditional SSRI vs. SNRI head-to-head RCTs and meta-analyses (e.g., comparisons in major depressive disorder) have used clinician-rated scales such as the Hamilton Depression Rating Scale (HDRS) or the Montgomery-Åsberg Depression Rating Scale (MADRS), as primary outcomes [36]. However, PHQ-9 is commonly used to monitor response in real-world cohorts where SSRIs and SNRIs are both prescribed [26]. Therefore, this study may contribute to demonstrating the value of PHQ-9 scores as a primary outcome for antidepressant effectiveness and quality improvement projects.

This study provides preliminary routine care evidence from Saudi clinical practice, where published comparative data on intranasal esketamine remain limited. It characterized the patients selected for esketamine treatment and reported symptoms and safety outcomes over 6 months. However, the study’s retrospective design, nonrandom treatment allocation, residual confounding, small sample size, incomplete follow-up, and reliance on medical record documentation constrain causal inference and generalizability. Prospective studies with prespecified outcome assessments, transparent handling of missing data, and adjusted analyses of binary clinical outcomes are needed.

Limitations: This study had several limitations. First, its retrospective observational design does not allow for causal inference. Treatment allocation was not randomized, and the groups differed in important baseline characteristics, including sex distribution, educational level, depressive symptom severity, and the number of failed antidepressant treatments. Although propensity score matching was applied to the continuous PHQ-9 outcomes, residual and unmeasured confounding remain possible. Second, outcome ascertainment depended on electronic medical record documentation, which may have resulted in missing data, variations in the timing of PHQ-9 assessments, and under-ascertainment of adverse events. Third, follow-up findings reflect outcomes among patients who remained in treatment or care and may be subject to attrition bias, particularly because discontinuation may have been related to a lack of response, tolerability, or patient preference. Fourth, detailed treatment-exposure data were incompletely captured in the retrospective records. Although esketamine was administered under healthcare-provider supervision and documented administrations indicate receipt of those individual doses, complete patient-level information on the number of administrations, cumulative dose, duration of exposure, and deviations from the intended induction or maintenance schedule was unavailable. Adherence to concomitant oral antidepressants and longitudinal changes in antidepressant and other psychotropic regimens could not be reliably determined. These limitations may have influenced the observed clinical outcomes and adverse-event patterns. Fifth, the classification of adverse events as serious was based on routine electronic medical record documentation rather than prespecified or independently adjudicated seriousness criteria. Information on event onset, duration, severity grade, clinical management, hospitalization, causal relationship, and dose relationship was incomplete. Accordingly, the reported serious events reflect documented clinical records and should not be interpreted as formally verified serious adverse events or as comparative estimates of treatment-related risk. Finally, the single-setting sample may limit the generalizability to other regions, private-sector settings, and patients with psychiatric or cardiovascular conditions excluded from this cohort.

## 5. Conclusions

In this routine care cohort of adults with TRD, intranasal esketamine was prescribed more often to patients with greater baseline depressive symptom severity and more extensive previous antidepressant nonresponse. After propensity score matching, follow-up PHQ-9 scores were modestly lower in the esketamine group at 3 and 6 months; however, the estimated differences were below the conventional individual-level MCID threshold. No remission was documented in either group. Given the nonrandom treatment allocation, baseline imbalance, and potential attrition bias, these findings are hypothesis-generating and should not be interpreted as evidence of a causal comparative benefit. Prospective, adequately powered studies are needed to assess comparative effectiveness and safety in routine clinical practice.

## Figures and Tables

**Table 1:** Baseline demographics and clinical characteristics.

Characteristic	Esketamine Nasal Spray+ Oral Antidepressants n = 82	Oral Antidepressant Venlafaxine+ Oral Antidepressants n = 87	SMD	Test Statistic	*p* Value
Age, mean (SD) [range], y	37.1 (10.5) [20–62]	36.6 (11.7) [18–73]	0.042	−0.270	0.786
BMI, mean (SD) [range]	27.1 (3.3) [20.1–40.1]	27.3 (1.6) [22–31.2]	0.371	2.38	0.017
Duration of disease (SD) [range], y	7.8 (7.2) [1–24]	7.8 (7.9) [1–26]	0.134	0.870	0.384
Sex					
Male	63 (76.8)	53 (60.9)	0.349	4.96	0.026
Female	19 (23.2)	34 (39.1)			
Nationality					
Saudi	81 (98.8)	87 (100)	0.157		0.485
Non-Saudi	1 (1.2)	0 (0)			
Marital status					
Married	51 (62.2)	59 (67.8)	0.099	0.604	0.573
Single	28 (34.1)	25 (28.7)			
Divorced	3 (3.7)	3 (3.4)			
Education level					
High school graduate or less	45 (54.9)	64 (73.6)	0.587		0.001
University degree	36 (43.9)	17 (19.5)			
Illiterate	1 (1.2)	6 (6.9)			
Occupation					
Employed	48 (58.5)	45 (51.7)	0.160	1.08	0.345
Unemployed	34 (41.6)	42 (48.3)			
Smoking status					
Non-smoker	67 (82.7)	69 (79.3)	0.051	0.113	0.287
Current smoker	9 (11.1)	11 (12.6)			
Previous smoker	5 (6.2)	7 (8.0)			
Family history of MDD					
Yes	21 (25.6)	30 (34.5)	0.359	5.32	0.070
No	37 (45.1)	44 (50.6)			
Unknown	24 (29.3)	13 (14.9)			
Baseline PHQ-9 score			0.601	14.15	0.001
15–19 (moderately severe depression)	30 (36.6)	57 (65.5)			
20–27 (severe depression)	52 (63.4)	30 (34.5)			
Number of previous failed AD. n (%)			0.999	5.71	0.001
2 failed	1 (1.2)	19 (21.8)			
3–4 failed	22 (26.8)	45 (51.7)			
5–6 failed	36 (43.9)	19 (21.8)			
7–8 failed	19 (23.2)	4 (4.6)			
9–10 failed	4 (4.9)	0 (0)			
Concurrent prescribed medications. n (%)					
SSRI	43 (52.4)	32 (36.8)			
Fluoxetine	21 (25.6)	7 (8.0)			
Escitalopram	8 (9.8)	10 (11.5)			
Paroxetine	8 (9.8)	4 (4.6)			
Sertraline	5 (6.1)	4 (4.6)			
SNRI	51 (62.2)	64 (73.3)			
Desvenlafaxine	12 (14.6)	11 (12.6)			
Duloxetine	7 (8.5)	7 (8.0)			
TCA	14 (17.1)	18 (20.7)			
Amitriptyline	7 (8.5)	15 (17.2)			
Clomipramine	7 (8.5)	3 (3.4)			
Atypical antidepressant	20 (24.4)	23 (26.4)			
Bupropion	13 (15.9)	5 (5.7)			
Mirtazapine	8 (9.8)	22 (25.3)			
Antipsychotics	50 (61.0)	54 (62.1))			
Quetiapine	26 (31.7)	35 (40.2)			
Aripiprazole	13 (15.9)	12 (13.8)			
Olanzapine	12 (14.6)	11 (12.6)			
Benzodiazepines	11 (13.4)	5 (5.7)			
Mood stabilizer	3 (3.7)	(3.4)			

AD, antidepressant; BMI, body mass index; MDD, major depressive disorder; PHQ-9, Patient Health Questionnaire-9; SMD, standardized mean difference; SNRI, serotonin–norepinephrine reuptake inhibitor; SSRI, selective serotonin reuptake inhibitor; TCA, tricyclic antidepressant. Categorical variables were compared using Pearson’s chi-square test or Fisher’s exact test, as appropriate. Age was compared using an independent samples *t*-test. BMI and duration of disease were compared using the Mann–Whitney U test. The test-statistic column reports t, Z, or $\chi^{2}$ as applicable; no test statistic is reported for Fisher’s exact test. SMDs describe baseline imbalance in the unmatched cohort. Concurrent psychotropic medication use is descriptive only; individual medications within a class are not mutually exclusive.

**Table 2:** Intended treatment schedules and initial prescribed doses.

Doses (mg)	Frequency (%)
Esketamine doses	
56	21 (25.7)
84	61 (74.3)
Dose frequency	Twice weekly (induction) then titrated
Venlafaxine doses	
75	14 (16.1)
150	56 (64.4)
225	17 (19.5)
Dose frequency	Once daily

Patient-level cumulative dose, number of administrations, and longitudinal changes in concomitant medications were not consistently available in the retrospective records.

**Table 3:** Unadjusted treatment outcomes at 28 days, 3 months, and 6 months among patients with treatment-resistant depression.

Outcome	Esketamine	Venlafaxine	Test Statistic	*p* Value
28-day induction phase			1.76	0.182
Continued treatment	61 (74.4)	72 (82.8)		
Discontinued treatment	21 (25.6)	15 (17.2)		
Reason for drug discontinuation				
Failure/No response	11 (13.4)	8 (9.2)	0.003	0.955
Patient preference/side effects	10 (12.2)	7 (8.0)		
Continuation phase	61 (74.4)	72 (82.8)		
Change in Follow-up PHQ-9 at 3 months				
Mean change (95% CI)	−4.73 (−5.37, −4.08)	−3.41 (−3.75, −3.07)	3.81	<0.001
Remission	0 (0)	0 (0)		0.001
Response	1 (1.6)	0 (0)		
MCID without response/remission	37 (60.7)	23 (31.9)		
Improvement below MCID	23 (37.7)	49 (68.1)		
Change in Follow-up PHQ-9 at 6 months				
Mean change (95% CI)	−8.83 (−9.76, −7.90)	−6.26 (−6.87, −5.64)	4.53	<0.001
Remission	0 (0)	0 (0)	16.87	0.001
Response	27 (44.3)	9 (12.5)		
MCID without response/remission	34 (55.7)	63 (87.5)		

CI, confidence interval; MCID, minimal clinically important difference; PHQ-9, Patient Health Questionnaire-9. Data are presented as n (%) unless otherwise indicated. Remission was defined as a follow-up PHQ-9 score < 5. Response was defined as a ≥50% reduction from baseline PHQ-9 score with a follow-up score < 10. MCID without response/remission was defined as an absolute PHQ-9 reduction of ≥5 points from baseline among patients who did not meet response or remission criteria. Patients classified as “Improvement below MCID” had a reduction of <5 points, no change, or worsening in PHQ-9 score. Percentages for each follow-up outcome were calculated using the number of patients who entered the maintenance phase with an observed PHQ-9 score at that time point, not the baseline cohort denominator. The denominators were: 3 months, esketamine n = 61 and venlafaxine n = 72; 6 months, esketamine n = 61 and venlafaxine n = 72. Categorical comparisons were unadjusted and are presented descriptively because of baseline imbalance, nonrandom treatment allocation, and follow-up attrition.

## Data Availability

Owing to ethical restrictions mandated by the Institutional Review Board of the Ministry of Health, the data supporting the findings of this study are available from the corresponding author upon reasonable request.

## Supplementary Materials

The following supporting information can be downloaded at: https://www.mdpi.com/article/10.3390/healthcare14172717/s1, Table S1. Standardized mean differences of baseline covariates before and after propensity score matching at 3 months and 6 months’ follow-up.

## Abbreviations

The following abbreviations are used in this manuscript:

AD	Antidepressant
BMI	Body mass index
MCID	Minimal clinically important difference
MDD	Major depressive disorder
PHQ-9	Patient Health Questionnaire-9
SNRI	Serotonin-Norepinephrine Reuptake Inhibitor
SSRI	Selective Serotonin Reuptake Inhibitor
TCA	Tricyclic Antidepressants
