# Supplementary material for: Real-World Effectiveness and Safety of Esketamine Nasal Spray in Treatment-Resistant Depression: A Retrospective Observational Study
Source: Healthcare (Basel). 2026 Aug 26;14(17):2717. doi: 10.3390/healthcare14172717 (PMC13565195; doi:10.3390/healthcare14172717)
Supplement: Supplementary file 1 [file healthcare-14-02717-s001.zip › healthcare-4483335-supplementary.pdf]

Supplementary Table S1. Standardized mean differences of baseline covariates before and after propensity score matching at 3 months and 6 months' follow-up.

**Covariate**	**3-month raw SMD**	**3-month matched SMD**	**6-month raw SMD**	**6-month matched SMD**
Age, y	0.026	0.053	−0.057	-0.089
BMI	−0.201	0.206	−0.196	0.061
Duration of disease, y	0.070	0.026	0.062	0.103
Sex	0.351	0.097	0.385	0.032
Marital status	−0.089	−0.047	−0.168	−0.051
Education level	0.571	−0.059	0.689	0.171
Occupation	0.215	0.126	0.267	0.082
Smoking status	−0.029	0.065	−0.031	0.096
Family history of MDD	0.206	−0.290	0.196	−0.249
Baseline PHQ-9 score	0.557	−0.123	0.538	0.068
Number of failed AD.	0.999	0.147	0.978	0.128
Concurrent prescribed psychotropics	0.715	0.046	0.693	0.023

Propensity score matching was 1:1 nearest neighbor with replacement, estimated separately in each outcome-specific complete-case sample, without a caliper.

An absolute standardized mean difference (SMD) <0.10 was considered to indicate acceptable balance. At 3 months, matching reduced imbalance for several covariates; however, residual imbalance remained for BMI (SMD = 0.206), number of failed AD (SMD = 0.147), and family history of MDD (SMD = 0.290). At 6 months, the largest residual imbalance was for family history of MDD (SMD = 0.249); education level showed a smaller residual imbalance (SMD = 0.171), whereas all remaining covariates had absolute SMDs ≤0.129.
